# MicroRNA expression profile associated with coronary heart disease in patients with resistant arterial hypertension

**DOI:** 10.3389/fmolb.2026.1905966

**Published:** 2026-09-10

**Authors:** Robert Tomasz Błaszczyk, Alicja Petniak, Jacek Bogucki, Janusz Kocki, Łukasz Wiśniowski, Andrzej Głowniak

**Affiliations:** 1 Department of Cardiology, Medical University of Lublin, Lublin, Poland; 2 Department of Clinical Genetics, Medical University of Lublin, Lublin, Poland; 3 Faculty of Medicine, John Paul II Catholic University of Lublin, Lublin, Poland

**Keywords:** coronary heart disease, microRNA, peripheral blood mononuclear cells, resistant arterial hypertension, type 2 diabetes

## Abstract

**Background:**

Resistant arterial hypertension (RAH) is associated with a high burden of cardiovascular complications, particularly coronary heart disease (CHD). MicroRNAs (miRNAs) are important regulators of cardiovascular remodeling and metabolic dysfunction; however, data regarding peripheral blood mononuclear cell (PBMC) miRNA expression in patients with RAH and concomitant CHD remain limited. This study aimed to evaluate PBMC miRNA expression profiles associated with CHD in patients with RAH and to explore their relationship with metabolic abnormalities.

**Methods:**

In this cross-sectional study, 115 patients with RAH were enrolled. PBMCs were isolated from peripheral blood and expression levels of selected microRNAs were quantified using TaqMan-based quantitative real-time PCR. Relative expression was calculated using the 2^−ΔΔCT^ method and expressed as log-transformed relative quantification (logRQ). Patients were stratified according to the presence of CHD. Correlation analyses, subgroup comparisons, and receiver operating characteristic (ROC) analyses were performed.

**Results:**

Compared with patients with isolated RAH, those with concomitant CHD exhibited significantly higher PBMC expression levels of miR-195 (p = 0.0148), miR-26b (p = 0.0166), miR-208 (p = 0.0435), and miR-320 (p = 0.0402). These microRNAs demonstrated strong positive intercorrelations, indicating a coordinated expression pattern. Within the CHD subgroup, expression levels of all four microRNAs were significantly higher in patients with type 2 diabetes (T2D) and positively correlated with glycated hemoglobin and body mass index. ROC analysis demonstrated modest discriminative ability for identifying T2D within the CHD subgroup, with the highest performance observed for miR-195 (AUC = 0.706).

**Conclusion:**

Patients with RAH and concomitant CHD exhibit a distinct PBMC microRNA expression profile characterized by increased expression of miR-195, miR-208, miR-26b, and miR-320. The association of these microRNAs with T2D and markers of metabolic dysfunction suggests that they may reflect the combined cardiovascular and metabolic disease burden in patients with advanced hypertension.

## 1 Introduction

Resistant arterial hypertension (RAH) represents a distinct and clinically important phenotype of arterial hypertension (AH), defined by inadequate blood pressure control despite the use of multiple antihypertensive agents and associated with a markedly increased risk of cardiovascular morbidity and mortality. Beyond persistent blood pressure elevation despite guideline-directed pharmacological therapy, RAH is characterized by complex pathophysiological mechanisms involving sustained activation of the renin–angiotensin–aldosterone system (RAAS), sympathetic nervous system overactivity, endothelial dysfunction, vascular remodeling, oxidative stress, and chronic low-grade inflammation. Increasing evidence indicates that immune dysregulation and inflammatory signaling are integral components of RAH pathogenesis, contributing not only to impaired blood pressure control but also to progressive target-organ damage affecting the heart, kidneys, and vasculature (Azizi et al., 2024; Schiffrin and Fisher, 2024; Yaacoub et al., 2024; He et al., 2025).

Coronary heart disease (CHD) is one of the most frequent and clinically relevant complications of RAH. The coexistence of RAH and CHD identifies a particularly high-risk population characterized by accelerated atherosclerosis, persistent endothelial dysfunction, myocardial remodeling, and an increased incidence of adverse cardiovascular events. Importantly, the relationship between RAH and CHD is bidirectional. Long-standing uncontrolled hypertension promotes coronary atherosclerosis and myocardial ischemia, whereas established CHD, particularly when accompanied by left ventricular dysfunction or heart failure, further aggravates neurohormonal activation and vascular dysfunction, thereby contributing to the persistence of resistant hypertension. Consequently, patients with concomitant RAH and CHD represent a clinically distinct phenotype in whom multiple cardiovascular and inflammatory pathways converge (Gallo and Savoia, 2024; Kuchmin et al., 2023; Pinto, 2020).

Although numerous studies have demonstrated altered microRNA expression in AH and CHD, the vast majority have focused on circulating cell-free microRNAs measured in plasma or serum because of their potential utility as minimally invasive biomarkers. However, circulating extracellular microRNAs represent only one component of disease-associated molecular alterations. Increasing evidence indicates that peripheral blood mononuclear cells (PBMCs), as transcriptionally active immune cells directly involved in chronic inflammation, immune activation, endothelial dysfunction, and vascular remodeling, provide complementary biological information regarding the mechanisms underlying cardiovascular disease. Consequently, PBMC-derived microRNA expression profiles may provide mechanistic insights into disease-associated immune and inflammatory processes that complement information obtained from circulating extracellular microRNAs (Sufianov et al., 2025; Searles, 2024; Gangwar et al., 2018; Abbas and Gaye, 2025).

Previous studies have identified numerous microRNAs associated with AH, CHD, myocardial infarction, heart failure, and metabolic disorders, supporting their potential as biomarkers of cardiovascular risk and disease progression. Several investigations have also demonstrated altered expression of selected PBMC-derived microRNAs in patients with AH or CHD, suggesting that immune cell-associated microRNA profiles reflect ongoing cardiovascular remodeling and inflammatory activity. However, available evidence remains limited and fragmented, with most studies evaluating either AH and CHD separately, frequently in heterogeneous patient populations and using different biological specimens. Moreover, the potential influence of concomitant coronary heart disease on PBMC-derived microRNA expression in patients with resistant arterial hypertension has not been specifically investigated. Consequently, it remains unclear whether concomitant coronary heart disease is associated with a distinct PBMC-derived microRNA expression profile in patients with RAH and whether such alterations are related to the high burden of metabolic comorbidities characteristic of this population (Kondracki et al., 2024; Yao et al., 2018; Kontaraki et al., 2015; Karlin et al., 2024).

Patients with RAH frequently present with metabolic comorbidities, particularly obesity and type 2 diabetes (T2D), which further amplify cardiovascular risk through shared mechanisms including chronic inflammation, oxidative stress, endothelial dysfunction, and insulin resistance. Emerging evidence suggests that several cardiovascular-associated microRNAs also participate in the regulation of glucose metabolism and metabolic homeostasis, indicating that alterations in miRNA expression may reflect the combined effects of cardiovascular and metabolic disease rather than isolated pathological processes. However, the interplay between CHD, metabolic abnormalities, and PBMC-derived microRNA expression has not been systematically investigated in patients with RAH. Elucidating these relationships may provide novel insights into the molecular interplay between cardiovascular and metabolic disease in RAH (Herrera et al., 2010; Ling et al., 2009; Xu et al., 2015; Rottiers and Näär, 2012).

Therefore, the aim of the present study was to characterize the PBMC-derived microRNA expression profile in patients with resistant arterial hypertension according to the presence of coronary heart disease. In addition, we investigated the relationships between CHD-associated microRNAs and selected clinical, metabolic, biochemical, and echocardiographic parameters. Because T2D frequently coexists with RAH and CHD and may influence microRNA expression, an exploratory subgroup analysis according to diabetes status was also performed in patients with concomitant CHD.

## 2 Patients and methods

### 2.1 Patients

The study included 115 patients with RAH treated at the Department of Cardiology, Medical University of Lublin, between January and December 2021. RAH was defined in accordance with current guidelines as uncontrolled blood pressure despite treatment with at least three antihypertensive agents of different classes, including a diuretic, or controlled blood pressure requiring four or more antihypertensive medications (Williams et al., 2018; Mancia et al., 2023).

CHD was diagnosed based on documented medical history, including prior myocardial infarction and/or coronary revascularization procedures (percutaneous coronary intervention or coronary artery bypass grafting), as well as documented stable coronary artery disease.

Hypercholesterolemia was defined as total cholesterol ≥190 mg/dL and/or LDL cholesterol ≥115 mg/dL. Hypertriglyceridemia was defined as fasting triglyceride concentration ≥150 mg/dL. Mixed dyslipidemia was defined as the coexistence of hypercholesterolemia and hypertriglyceridemia.

The study was conducted in accordance with the Declaration of Helsinki and was approved by the Bioethics Committee of the Medical University of Lublin (approval No. KE-0254/141/2020). Written informed consent was obtained from all participants prior to enrollment.

### 2.2 Blood collection and isolation of peripheral blood mononuclear cells

Peripheral venous blood was drawn into 2.7-mL S-Monovette tubes containing K3-EDTA (Sarstedt, Germany). For recovery of peripheral blood mononuclear cells (PBMCs), freshly collected blood was combined with an equal volume of calcium- and magnesium-free phosphate-buffered saline (PBS; Biomed-Lublin, Poland). The diluted material was gently layered onto 3 mL of Ficoll-Paque Plus (Cytiva, Uppsala, Sweden) in sterile 15-mL conical tubes and centrifuged at 400 × g for 30 min at 20 °C with the brake disengaged using a 5810 R centrifuge (Eppendorf, Germany).

The PBMC-containing interface was transferred into clean tubes and washed twice in PBS by centrifugation at 450 × g for 10 min at 20 °C. Following the washing steps, cells were resuspended in 1 mL of PBS, divided into sterile 1.5-mL DNA LoBind tubes (Eppendorf, Germany), and centrifuged at 400 × g for 10 min at 4 °C using a 5415 R centrifuge (Eppendorf, Germany). After removal of the supernatant, the PBMC pellets were stored at −80 °C until RNA extraction.

### 2.3 Total RNA extraction and reverse transcription

Total RNA, including the small RNA fraction, was isolated from frozen PBMC pellets with the mirVana™ miRNA Isolation Kit (Invitrogen, Thermo Fisher Scientific, Vilnius, Lithuania) in accordance with the manufacturer’s protocol. RNA yield and purity were evaluated using a NanoDrop 2000c spectrophotometer (Thermo Fisher Scientific, Waltham, MA, United States). All samples included in downstream analyses had A260/A280 ratios ranging from 1.8 to 2.0.

For each miRNA target, complementary DNA (cDNA) was generated using miRNA-specific stem-loop primers and the TaqMan™ MicroRNA Reverse Transcription Kit (Applied Biosystems, Vilnius, Lithuania). The 15-µL reverse transcription reaction contained 5 µL of RNA template corresponding to 10 ng of total RNA, 3 µL of 5× RT primer, 1.5 µL of 10× Reverse Transcription Buffer, 0.15 µL of 100 mM dNTP mix with dTTP, 1 µL of MultiScribe™ Reverse Transcriptase (50 U/µL), 0.19 µL of RNase inhibitor (20 U/µL), and 4.16 µL of nuclease-free water.

Reverse transcription was conducted on a Veriti Dx Thermal Cycler (Applied Biosystems, Foster City, CA, United States) using the following program: 30 min at 16 °C, 30 min at 42 °C, and 5 min at 85 °C. The generated cDNA was subsequently used as a template for quantitative PCR.

### 2.4 Quantitative real-time PCR and relative miRNA expression analysis

PBMC miRNA expression was determined using commercially available TaqMan™ MicroRNA Assays together with TaqMan™ Universal Master Mix II with UNG (Applied Biosystems, Vilnius, Lithuania) on a StepOnePlus™ Real-Time PCR System (Applied Biosystems, Waltham, MA, United States). These assays contain manufacturer-designed and experimentally validated stem-loop reverse transcription primers and sequence-specific TaqMan primer/probe sets; therefore, no additional in-house primer validation was performed. Each 10-µL amplification mixture consisted of 0.67 µL of cDNA, 0.5 µL of the miRNA-specific primer/probe assay, 5 µL of master mix, and 3.83 µL of nuclease-free water. Amplification was performed in triplicate in 96-well MicroAmp™ Fast Optical 0.1-mL reaction plates.

The predefined miRNA panel comprised the following commercially validated TaqMan™ MicroRNA Assays: hsa-miR-1 (assay ID 002222), hsa-miR-21 (assay ID 000397), hsa-miR-26b (assay ID 000407), hsa-miR-126* (assay ID 000451), hsa-miR-133a (assay ID 002246), hsa-miR-143 (assay ID 002249), hsa-miR-145* (assay ID 002149), hsa-miR-155 (assay ID 002623), hsa-miR-195 (assay ID 000494), hsa-miR-208 (assay ID 000511), and hsa-miR-320 (assay ID 002277). RNU48/SNORD48 (assay ID 001006) was used as the endogenous reference for normalization of PBMC-derived microRNA expression. RNU48 is a commonly used endogenous control for miRNA expression studies performed in cellular material, including PBMCs, due to its relatively stable expression in nucleated blood cells (Atarod et al., 2014; Masè et al., 2017; Błaszczyk et al., 2024; Błaszczyk et al., 2026).

The thermal cycling protocol comprised an initial incubation for 2 min at 50 °C, polymerase activation for 10 min at 95 °C, followed by 40 cycles of denaturation at 95 °C for 15 s and annealing/extension at 60 °C for 60 s. Relative expression levels were determined using the comparative Ct method (RQ = 2^−ΔΔCt), with the mean ΔCt of the healthy control group serving as the calibrator, as described by Livak and Schmittgen (Livak and Schmittgen, 2001). The healthy control group used for calibration consisted of 33 individuals. RQ values were log-transformed (logRQ) prior to statistical analysis, and calculations were performed with ExpressionSuite Software version 1.0.3 (Life Technologies).

The laboratory workflow for PBMC processing and TaqMan-based miRNA quantification was based on the protocol applied in our previous RAH study; the present investigation used the miRNA targets listed above (Błaszczyk et al., 2024; Błaszczyk et al., 2026).

Samples with unavailable or technically inadequate qPCR measurements for a given microRNA were excluded only from analyses involving that specific target. No imputation of missing values was performed.

### 2.5 Statistical analysis

Statistical analyses were performed using Statistica software (version 13.3). The distribution of continuous variables was assessed using the Shapiro–Wilk test. Because most variables showed non-normal distributions, continuous data are presented as median and interquartile range (IQR), whereas categorical variables are presented as counts and percentages.

Comparisons of microRNA expression levels between independent groups were performed using the Mann–Whitney U test. Correlations between microRNA expression levels and clinical, biochemical, and echocardiographic parameters were assessed using Spearman’s rank correlation coefficient. Intercorrelations among CHD-associated microRNAs were assessed in the overall RAH cohort, whereas correlations between these microRNAs and BMI, HbA1c, and fasting glucose were evaluated within the CHD subgroup. Differences in microRNA expression according to the presence of T2D within the CHD subgroup were assessed using the Mann–Whitney U test.

Receiver operating characteristic (ROC) curve analysis was performed to evaluate the discriminative ability of selected microRNAs for identifying T2D among patients with CHD. The area under the ROC curve (AUC) with 95% confidence intervals (95% CI) was calculated for each microRNA. Statistical significance of AUC values was assessed using the z test against the null hypothesis of AUC = 0.50.

All statistical tests were two-sided, and a p value < 0.05 was considered statistically significant.

The primary analysis compared PBMC microRNA expression levels between patients with RAH with and without concomitant CHD. Secondary exploratory analyses included comparisons according to T2D status within the CHD subgroup, correlation analyses between CHD-associated microRNAs and selected clinical, metabolic, biochemical, and echocardiographic variables, as well as ROC curve analyses evaluating the ability of CHD-associated microRNAs to discriminate T2D status.

Because of the exploratory nature of the study and the predefined candidate microRNA panel, no formal adjustment for multiple testing was applied. Therefore, all reported p values should be interpreted as exploratory and hypothesis-generating.

## 3 Results

### 3.1 Characteristics of study population

The analyzed cohort consisted of 115 patients with RAH (54% women), including a substantial proportion with documented CHD (69.6%). The median age was 66 years (IQR 61–73), and the median body mass index (BMI) was 29.8 kg/m^2^ (IQR 27.2–33.2). The cohort was characterized by long-standing AH, with a median duration exceeding 10 years, and a median duration of RAH of approximately 4 years. The median total cholesterol concentration was 200 mg/dL, the median LDL-cholesterol concentration was 135 mg/dL, and the median HDL-cholesterol concentration was 45 mg/dL. The median triglyceride concentration was 105 mg/dL (Table 1).

**TABLE 1:** Baseline demographic and clinical characteristics of the study population, including lipid profile parameters.

Variable	Value (n = 115)
Age, [years]	66 (61–73)
Women, n [%]	62 (53.9)
Body mass index [kg/m^2^]	29.8 (27.2–33.2)
Duration of AH [years]	10.0 (7.0–14.0)
Duration of RAH [years]	4.0 (3.0–5.0)
Total cholesterol [mg/dL]	200 (170–225)
LDL-cholesterol [mg/dL]	135 (102–154)
HDL-cholesterol [mg/dL]	45 (39–53)
Triglycerides [mg/dL]	105 (87–162)

Data are presented as median and IQR, or number (percentage), as appropriate. Lipid concentrations are expressed in mg/dL. AH, arterial hypertension; BMI, body mass index; IQR, interquartile range; RAH, resistant arterial hypertension.

Regarding categorical variables, previous myocardial infarction was present in 14.8% of patients. A history of coronary artery bypass graft (CABG) was reported in 7.8% of patients, while percutaneous coronary intervention (PCI) had been performed in 20.9% of patients. Overall, CHD defined on the basis of documented stable coronary artery disease, previous myocardial infarction, and/or coronary revascularization, was present in 69.6% of patients.

Despite the relatively low proportion of patients with prior myocardial infarction or revascularization procedures, the high prevalence of CHD indicates that the study population represents a cardiovascular high-risk cohort, even in the absence of frequent prior acute ischemic events. Metabolic comorbidities were common. Hypercholesterolemia was observed in 69.6% of patients, hypertriglyceridemia in 37.4% of patients, and mixed dyslipidemia in 31.3% of patients (Table 2).

**TABLE 2:** Prevalence of cardiovascular comorbidities and metabolic disorders in the study population.

Variable	N (%)
Previous myocardial infarction	17 (14.8)
Previous percutaneous coronary intervention (PCI)	24 (20.9)
Previous coronary artery bypass graft (CABG)	9 (7.8)
Coronary heart disease	80 (69.6)
Hypercholesterolemia	80 (69.6)
Hypertriglyceridemia	43 (37.4)
Mixed dyslipidemia	36 (31.3)

Data are presented as number of patients and percentage of the total study population.

PBMC microRNA expression levels, expressed as log-transformed relative quantification (logRQ), demonstrated substantial inter-individual variability across the analyzed panel (Table 3). Median expression values for most microRNAs were close to zero, as expected for normalized qPCR data; however, wide IQRs indicated considerable biological heterogeneity.

**TABLE 3:** PBMC microRNA expression levels in patients with resistant arterial hypertension.

microRNA	N	Median (IQR)
hsa-miR-1	103	0.23 (−0.39–0.78)
hsa-miR-126*	105	0.28 (−0.18–0.79)
hsa-miR-133a	106	−0.01 (−0.55–0.67)
hsa-miR-143	106	0.32 (−0.53–1.04)
hsa-miR-145*	104	0.60 (−0.28–1.32)
hsa-miR-155	106	0.49 (0.10–0.99)
hsa-miR-195	106	0.25 (−0.34–0.87)
hsa-miR-208	104	0.84 (0.25–1.46)
hsa-miR-21	106	0.72 (0.29–1.28)
hsa-miR-26b	106	0.42 (0.08–0.91)
hsa-miR-320	106	−0.07 (−0.52–0.59)

MicroRNA expression levels are shown as log-transformed relative quantification (logRQ) calculated using the 2^−ΔΔCT^ method.

### 3.2 Correlation between PBMC microRNA expression levels and selected clinical variables

**FIGURE 1:**
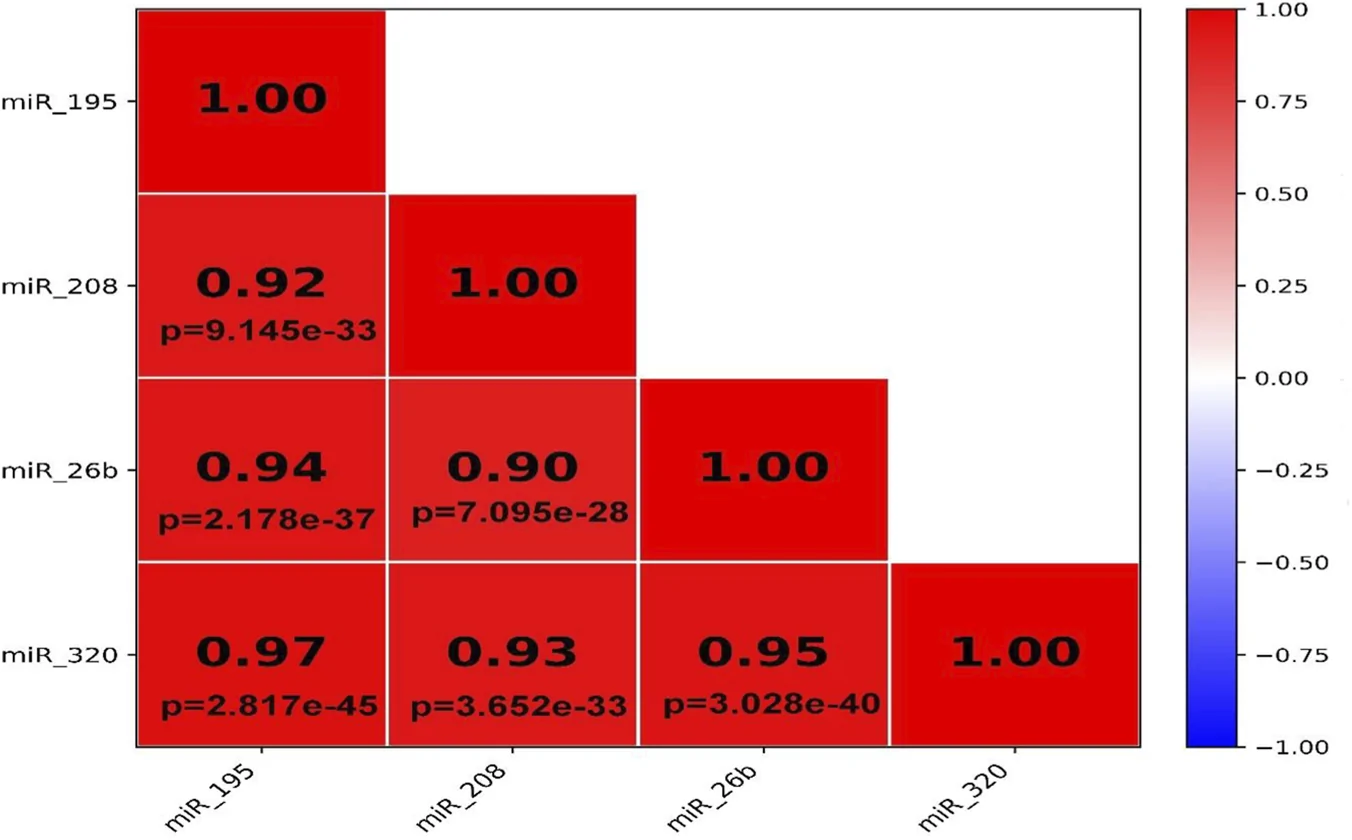
Co-expression pattern of CHD-associated PBMC microRNAs in patients with resistant arterial hypertension. Spearman correlation matrix demonstrating strong positive correlations among miR-195, miR-208, miR-26b, and miR-320 in the overall RAH cohort. Pairwise analyses included patients with available measurements for both microRNAs. Correlation coefficients and p values are displayed within each cell.

In the overall RAH cohort, the four microRNAs subsequently identified as CHD-associated demonstrated a highly coordinated expression pattern. Strong positive correlations were observed among miR-195, miR-208, miR-26b, and miR-320, with Spearman correlation coefficients ranging from 0.90 to 0.97 (Figure 1). A broader overview of correlations between clinical variables and the analyzed PBMC microRNAs is presented in Supplementary Figure S1.

Within the CHD subgroup, all four microRNAs demonstrated positive correlations with BMI and HbA1c. Significant positive correlations with fasting glucose were observed for miR-195 and miR-320, whereas those for miR-208 and miR-26b were not statistically significant (Table 4). Correlations between PBMC microRNAs, echocardiographic parameters, and lipid profile variables are shown in Supplementary Figure S2.

**TABLE 4:** Correlations between CHD-associated PBMC microRNAs and metabolic parameters in patients with coronary heart disease.

Variable	miR-195	miR-208	miR-26b	miR-320
BMI (kg/m^2^)	r = 0.281; p = 0.0140	r = 0.262; p = 0.0224	r = 0.284; p = 0.0128	r = 0.271; p = 0.0178
Glycated hemoglobin (HbA1c) (%)	r = 0.326; p = 0.0040	r = 0.317; p = 0.0053	r = 0.285; p = 0.0124	r = 0.303; p = 0.0079
Fasting glucose (mg/dL)	r = 0.267; p = 0.0197	r = 0.206; p > 0.05	r = 0.189; p > 0.05	r = 0.240; p = 0.0371

Values are Spearman rank correlation coefficients and corresponding p values. BMI, body mass index; HbA1c, glycated hemoglobin.

### 3.3 PBMC microRNA expression according to CHD status

#### 3.3.1 Previous myocardial infarction, PCI, and CABG

No statistically significant differences in microRNA expression were observed between patients with RAH and a history of myocardial infarction (n = 17) and those without prior myocardial infarction. Similarly, no significant differences were detected in comparisons based on prior PCI (n = 24) or CABG (n = 9). Although these subgroup analyses were limited by relatively small sample sizes, the findings suggest that isolated historical ischemic events or previous revascularization procedures were not the primary determinants of PBMC microRNA expression in this cohort.

Detailed results of analyses according to previous myocardial infarction, PCI, and CABG are provided in Supplementary Table S1.

#### 3.3.2 Coronary heart disease

**FIGURE 2:**
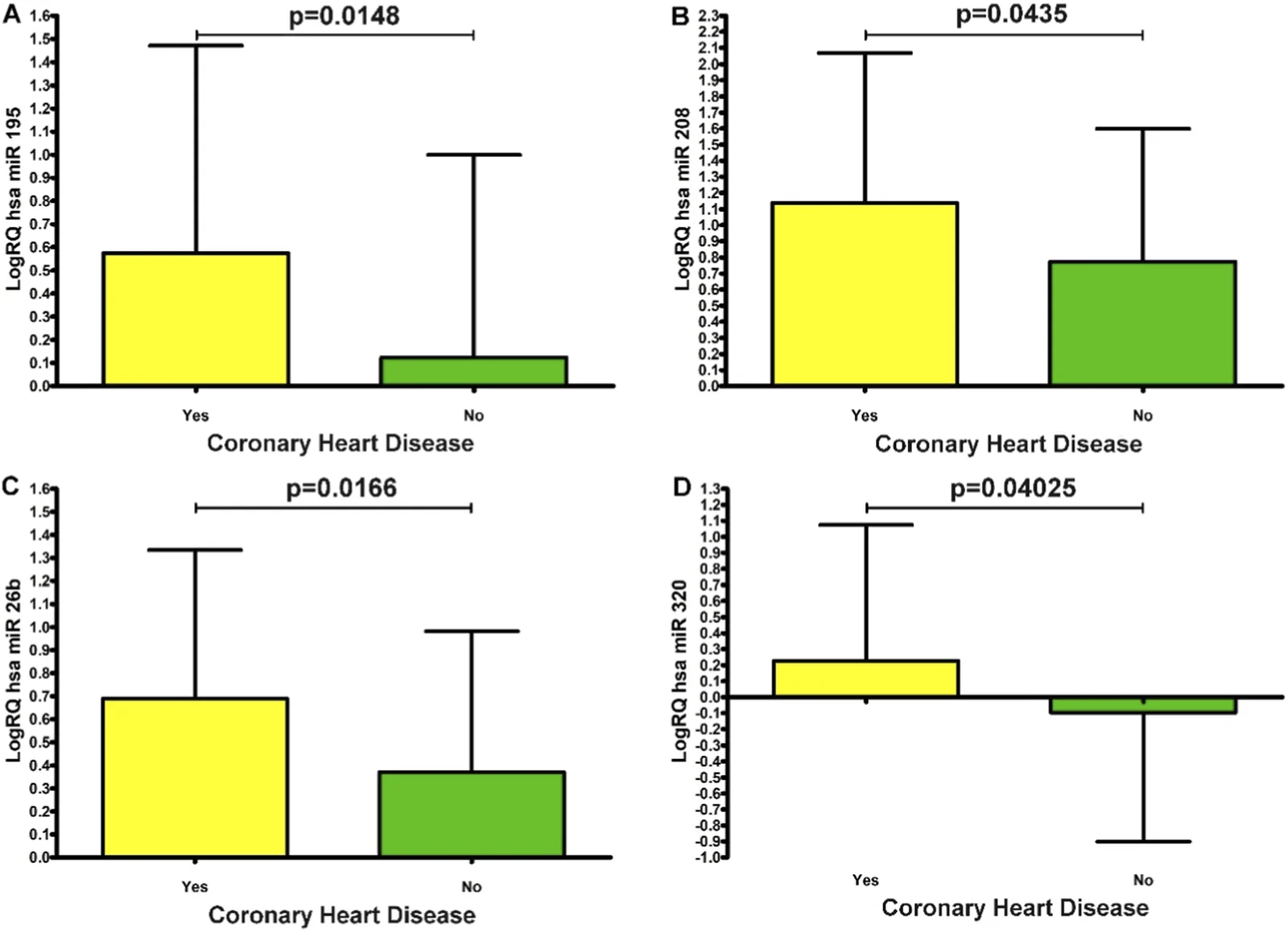
Expression of CHD-associated PBMC microRNAs in patients with resistant arterial hypertension according to coronary heart disease status **(A)** miR-195 **(B)** miR-208 **(C)** miR-26b, and **(D)** miR-320. The study groups comprised patients with CHD (n = 80) and without CHD (n = 35); analyses included patients with available measurements for each microRNA. Patients with CHD demonstrated significantly higher expression levels of all four microRNAs. Data are presented as median and interquartile range. P values were calculated using the Mann–Whitney U test.

In contrast, when patients with documented CHD were analyzed as a single group (n = 80), several statistically significant differences in PBMC microRNA expression were identified. In patients with RAH and concomitant CHD, PBMC expression levels of miR-195, miR-26b, miR-208, and miR-320 were significantly higher compared with patients with isolated RAH (*p* = 0.0148, 0.0166, 0.0435, and 0.0402, respectively) (Figure 2). In addition, several microRNAs showed borderline statistical significance, including hsa-miR-21 (*p* = 0.0533), hsa-miR-143 (*p* = 0.0626), hsa-miR-126* (*p* = 0.0710), hsa-miR-133a (*p* = 0.0814), and hsa-miR-145* (*p* = 0.0897).

### 3.4 Type 2 diabetes and CHD-associated microRNAs

To further explore factors associated with the identified CHD-related microRNA signature, patients with CHD were stratified according to the presence of T2D.

**FIGURE 3:**
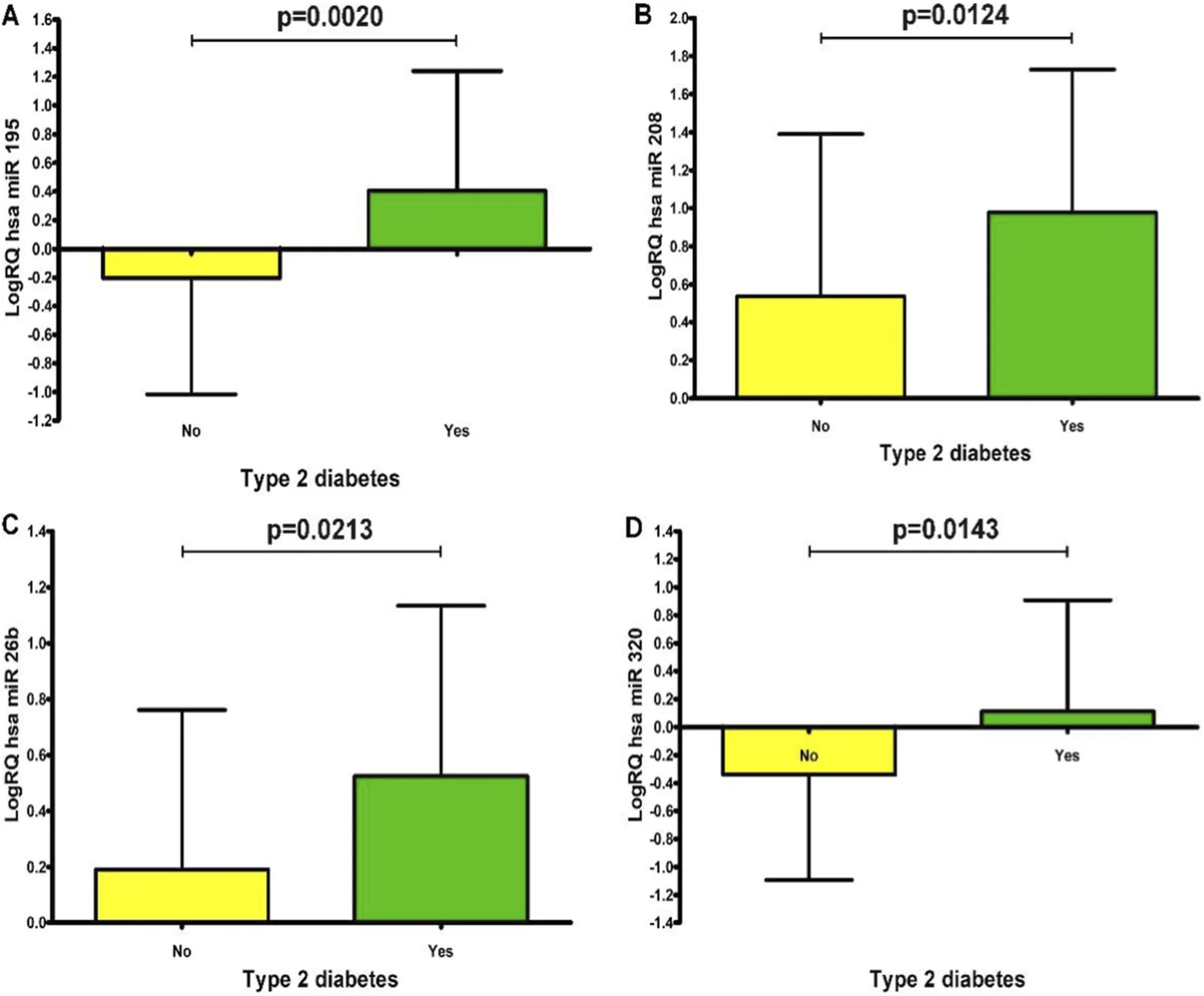
Expression of CHD-associated PBMC microRNAs according to type 2 diabetes status in patients with coronary heart disease **(A)** miR-195 **(B)** miR-208 **(C)** miR-26b, and **(D)** miR-320. The CHD subgroup comprised patients with T2D (n = 43) and without T2D (n = 37); analyses included patients with available measurements for each microRNA. Patients with T2D demonstrated significantly higher expression levels of all four microRNAs. Data are presented as median and interquartile range. P values were calculated using the Mann–Whitney U test.

Among patients with CHD, expression levels of miR-195, miR-208, miR-26b, and miR-320 were significantly higher in individuals with T2D compared with non-diabetic patients (Table 5; Figure 3). The observed differences reached statistical significance for all four microRNAs, including miR-195 (p = 0.0020), miR-208 (p = 0.0124), miR-26b (p = 0.0213), and miR-320 (p = 0.0143). Notably, all components of the identified CHD-associated microRNA signature demonstrated a consistent relationship with diabetic status. The most pronounced numerical between-group difference was observed for miR-195. These findings are consistent with the positive correlations of all four microRNAs with BMI and HbA1c and of miR-195 and miR-320 with fasting glucose.

**TABLE 5:** Expression of CHD-associated PBMC microRNAs according to type 2 diabetes status in patients with coronary heart disease.

MicroRNA	T2D (n = 43)	No T2D (n = 37)	p value
miR-195	0.40 (−0.26–1.02)	−0.39 (−0.73–0.22)	0.0020
miR-208	1.03 (0.40–1.52)	0.45 (−0.04–0.95)	0.0124
miR-26b	0.42 (0.05–0.97)	0.14 (−0.16–0.46)	0.0213
miR-320	0.01 (−0.45–0.74)	−0.43 (−0.81–0.04)	0.0143

Data are presented as median (interquartile range). P values were calculated using the Mann–Whitney U test. CHD, coronary heart disease; PBMC, peripheral blood mononuclear cells; T2D, type 2 diabetes.

### 3.5 ROC analysis of CHD-associated microRNAs for identification of type 2 diabetes

**FIGURE 4:**
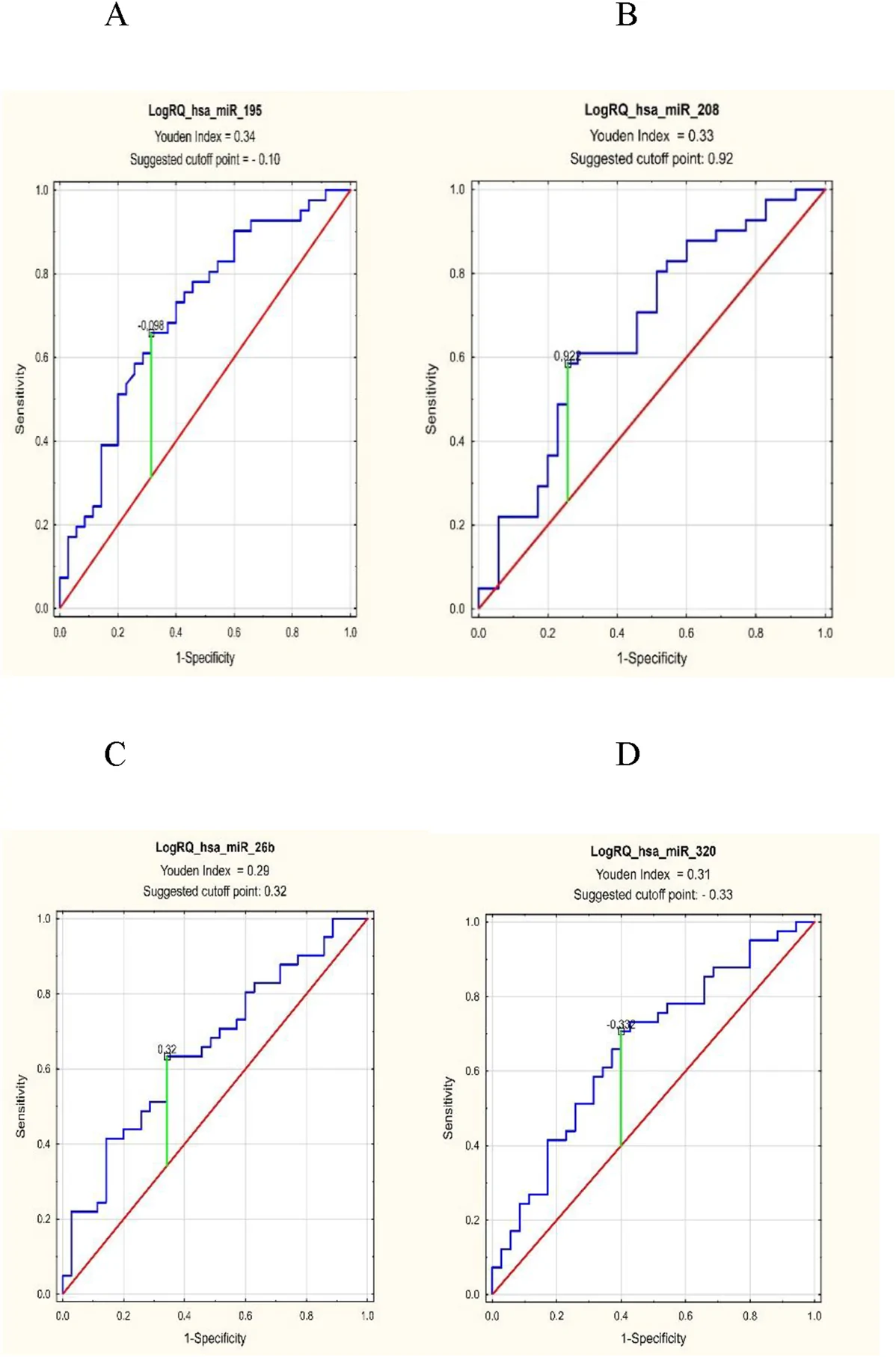
Receiver operating characteristic curves of CHD-associated PBMC microRNAs for identification of T2D in patients with CHD **(A)** miR-195 **(B)** miR-208 **(C)** miR-26b, and **(D)** miR-320. The highest discriminative performance was observed for miR-195 (AUC = 0.706).

Receiver operating characteristic (ROC) analysis was performed to evaluate the ability of CHD-associated microRNAs to discriminate between patients with and without T2D within the CHD subgroup (Figure 4; Table 6).

**TABLE 6:** Discriminative performance of CHD-associated PBMC microRNAs for identification of type 2 diabetes in patients with coronary heart disease.

MicroRNA	AUC	95% CI	P value
miR-195	0.706	0.588–0.824	0.0006
miR-208	0.668	0.544–0.791	0.0078
miR-26b	0.654	0.532–0.777	0.0137
miR-320	0.664	0.542–0.787	0.0087

Data are presented as area under the ROC curve (AUC) with corresponding 95% confidence intervals (95% CI). P values were calculated using the z test against the null hypothesis of AUC = 0.50. CHD, coronary heart disease; PBMC, peripheral blood mononuclear cell; ROC, receiver operating characteristic; AUC, area under the curve; CI, confidence interval.

All four CHD-associated microRNAs demonstrated statistically significant discriminatory ability for identifying T2D. Among the analyzed microRNAs, miR-195 showed the highest discriminative performance, with an area under the curve (AUC) of 0.706 (95% CI: 0.588–0.824; p = 0.0006). Modest discriminative ability was also observed for miR-208 (AUC = 0.668; 95% CI: 0.544–0.791; p = 0.0078), miR-320 (AUC = 0.664; 95% CI: 0.542–0.787; p = 0.0087), and miR-26b (AUC = 0.654; 95% CI: 0.532–0.777; p = 0.0137).

The relatively similar AUC values observed across the four microRNAs may be related to their strong coordinated expression pattern.

Overall, the identified PBMC microRNAs demonstrated modest but statistically significant ability to discriminate diabetic status among patients with CHD.

## 4 Discussion

The present study demonstrates that concomitant CHD is associated with a distinct PBMC-derived microRNA expression profile in patients with RAH. Compared with patients with isolated RAH, individuals with CHD exhibited significantly higher expression of miR-195, miR-208, miR-26b, and miR-320. These microRNAs demonstrated strong positive intercorrelations, indicating a coordinated expression pattern. Moreover, within the CHD subgroup, expression of all four microRNAs was significantly higher in patients with T2D and positively correlated with HbA1c and BMI. Although ROC analysis showed only modest discriminative performance, particularly for miR-195, the consistent associations observed across cardiovascular and metabolic phenotypes suggest that this PBMC-derived microRNA signature reflects the combined burden of CHD and metabolic dysfunction in patients with advanced resistant hypertension.

Unlike plasma- or serum-derived circulating microRNAs, PBMC-derived microRNAs originate from transcriptionally active immune cells that actively participate in chronic inflammation, endothelial dysfunction, vascular remodeling, and immune-mediated cardiovascular injury. Consequently, PBMC-derived microRNA profiles provide mechanistic information regarding cellular regulatory pathways involved in cardiovascular disease rather than solely reflecting extracellular biomarker release. Increasing evidence suggests that immune cell-derived microRNAs contribute to intercellular communication and may participate in the regulation of inflammatory and remodeling processes underlying RAH and CHD. Therefore, the use of PBMCs in the present study provides complementary biological insight into disease-associated molecular mechanisms and may facilitate a better understanding of the interaction between cardiovascular and metabolic dysfunction in patients with RAH (Sufianov et al., 2025; Searles, 2024; Abbas and Gaye, 2025; Atarod et al., 2014).

CHD emerged as the principal clinical factor associated with altered PBMC-derived microRNA expression in the present cohort. Patients with RAH and concomitant CHD consistently demonstrated higher expression of miR-195, miR-208, miR-26b, and miR-320 than patients without CHD. The coordinated upregulation of all four microRNAs suggests that CHD is associated with a distinct PBMC-derived molecular signature in patients with RAH rather than isolated dysregulation of individual microRNAs. This observation is consistent with previous studies demonstrating disease-specific PBMC microRNA expression profiles in patients with CHD, supporting the concept that immune cell-derived microRNAs are closely linked to the molecular mechanisms underlying atherosclerosis and coronary artery disease (Kondracki et al., 2024; Hoekstra et al., 2010; Sanlialp et al., 2020; Saadatian et al., 2023). Rather than reflecting blood pressure elevation alone, the observed PBMC microRNA profile appears to capture the additional molecular burden associated with concomitant CHD in patients with RAH.

An additional finding of the present study was that the observed PBMC-derived microRNA signature was associated with the presence of CHD as a chronic clinical condition rather than with individual manifestations of CHD, such as previous myocardial infarction, percutaneous coronary intervention, or coronary artery bypass grafting. Importantly, the observed differences were not explained by a previous history of myocardial infarction or coronary revascularization, as no significant differences in PBMC-derived microRNA expression were detected according to prior MI, PCI, or CABG. Instead, the identified PBMC-derived microRNA signature appears to reflect the cumulative biological burden of chronic coronary artery disease rather than the legacy of isolated ischemic events or previous revascularization procedures.

This interpretation is supported by previous studies demonstrating that microRNA expression changes dynamically throughout the natural course of CHD. Several circulating microRNAs exhibit marked alterations during acute coronary syndromes but subsequently normalize or undergo further modulation during the chronic phase of the disease, indicating that microRNA profiles depend on the underlying biological activity rather than solely on previous clinical events (de Gonzalo-Calvo et al., 2019; Stojkovic and Wojta, 2024; Kaur et al., 2020). Consequently, a history of myocardial infarction or coronary revascularization may not accurately reflect the current molecular processes associated with chronic CHD.

These observations prompted a more detailed evaluation of the biological and clinical relevance of the individual microRNAs associated with CHD in patients with resistant hypertension. Among the four differentially expressed microRNAs, miR-195 demonstrated one of the most consistent associations with both cardiovascular and metabolic characteristics. PBMC-derived miR-195 expression was significantly higher in patients with concomitant CHD and was further increased in those with T2D. Moreover, miR-195 correlated positively with HbA1c and BMI, suggesting that its expression reflects not only the presence of CHD but also the degree of metabolic impairment.

These findings extend our previous observations in patients with RAH, in whom increased PBMC-derived miR-195 expression was likewise associated with concomitant T2D (Błaszczyk et al., 2024). Considered together, these studies suggest that PBMC-derived miR-195 is consistently associated with increasing cardiometabolic complexity in resistant hypertension rather than with a single accompanying clinical condition.

Experimental studies have demonstrated that miR-195 regulates pathways involved in cardiomyocyte apoptosis, oxidative stress, and myocardial remodeling, including through the downregulation of Sirt1 (Zhu et al., 2011). Furthermore, dysregulated miR-195 expression has been implicated in diabetic cardiovascular complications and coronary artery disease, providing biological support for the associations observed in the present cohort (Szydełko and Matyjaszek-Matuszek, 2023; Caporali et al., 2024).

Rather than representing a marker of CHD alone, PBMC-derived miR-195 may reflect the cumulative effects of chronic cardiovascular injury and metabolic dysregulation in patients with resistant arterial hypertension. The consistent association of miR-195 with CHD, T2D, HbA1c, and BMI across our studies supports the hypothesis that PBMC-derived miR-195 may represent a marker of progressive cardiometabolic burden in patients with RAH.

Unlike miR-195, which showed strong associations with both cardiovascular and metabolic characteristics, miR-208 is regarded as a cardiac-enriched microRNA closely linked to myocardial remodeling and cardiomyocyte stress. In the present study, PBMC-derived miR-208 expression was significantly higher in patients with RAH and concomitant CHD and was further increased in those with T2D, suggesting that its expression may reflect chronic myocardial alterations accompanying advanced cardiovascular disease rather than previous ischemic events alone. Experimental and clinical studies have demonstrated that miR-208 regulates pathways involved in cardiac hypertrophy, fibrosis, and ventricular remodeling, and increased expression has been reported in several cardiovascular disorders, including coronary artery disease and heart failure (Zhao et al., 2020; González et al., 2022). Although miR-208 has been extensively investigated as a biomarker of acute myocardial injury, accumulating evidence indicates that its biological functions extend beyond acute ischemia and include long-term regulation of myocardial structure and function (Caporali et al., 2024). The present findings suggest that PBMC-derived miR-208 may reflect chronic myocardial remodeling associated with coronary artery disease and metabolic dysfunction rather than the consequences of a previous ischemic event alone.

In contrast to miR-208, which is primarily linked to myocardial remodeling, miR-26b has been implicated in the regulation of endothelial homeostasis, vascular inflammation, and atherosclerotic vascular remodeling. In the present study, PBMC-derived miR-26b expression was significantly increased in patients with RAH and concomitant CHD and was further elevated in those with T2D. Moreover, miR-26b expression correlated positively with HbA1c and BMI, indicating that vascular and metabolic abnormalities may jointly contribute to its dysregulation in patients with RAH.

Previous studies have demonstrated that miR-26b plays an important role in maintaining endothelial integrity and regulating inflammatory pathways involved in atherosclerosis and vascular remodeling. Recent experimental evidence has shown that loss of miR-26b accelerates atherosclerotic plaque development, enhances endothelial activation, and promotes vascular inflammation, whereas restoration of miR-26b expression attenuates these processes, supporting its atheroprotective role. Clinical studies have likewise associated altered miR-26b expression with coronary artery disease and metabolic disorders (Xu et al., 2015; Rottiers and Näär, 2012; Caporali et al., 2024; Peters et al., 2025). The increased PBMC expression observed in the present study may therefore represent a compensatory response to ongoing vascular inflammation rather than a direct proatherogenic effect.

Because PBMCs participate in vascular inflammation and atherosclerotic plaque development, altered miR-26b expression in these cells may reflect systemic immune mechanisms accompanying chronic CHD. Its additional increase in patients with T2D and positive correlations with HbA1c and BMI suggest that metabolic disturbances may further modulate these vascular and immune responses.

Among the investigated microRNAs, miR-320 has been consistently linked to metabolic regulation and vascular complications associated with diabetes. In the present study, PBMC-derived miR-320 expression was significantly higher in patients with RAH and concomitant CHD and was further increased in those with T2D. In addition, miR-320 expression showed positive correlations with both HbA1c and BMI, supporting a close relationship between this microRNA and adverse metabolic status.

Previous experimental and clinical studies have demonstrated that miR-320 regulates multiple pathways involved in insulin signaling, endothelial function, oxidative stress, and microvascular homeostasis. Altered miR-320 expression has been associated with insulin resistance, diabetic vascular complications, and coronary artery disease, suggesting that this microRNA integrates metabolic and vascular signals contributing to cardiovascular injury (Szydełko and Matyjaszek-Matuszek, 2023; Caporali et al., 2024; Li et al., 2021; Li et al., 2023; An et al., 2023).

The present findings are consistent with these observations and indicate that increased PBMC-derived miR-320 expression reflects the coexistence of chronic coronary artery disease and metabolic dysfunction rather than isolated hypertension. The additional increase observed in patients with T2D further supports the concept that miR-320 may serve as a marker of cardiometabolic burden in patients with resistant hypertension.

Although each of the investigated microRNAs is involved in distinct biological pathways, their coordinated upregulation in patients with RAH and concomitant CHD suggests that PBMC-derived microRNA expression reflects the cumulative burden of chronic cardiovascular disease rather than isolated pathological processes. While miR-208 is predominantly associated with myocardial remodeling, miR-26b and miR-320 appear to reflect vascular and metabolic dysfunction, whereas miR-195 integrates multiple aspects of cardiometabolic regulation. Overall, these findings indicate that PBMC-derived microRNA expression captures the complex interplay between myocardial remodeling, vascular dysfunction, and metabolic disturbances in patients with RAH.

The influence of T2D on PBMC-derived microRNA expression represents another important finding of the present study. Patients with concomitant T2D exhibited significantly higher expression of all four investigated microRNAs compared with those without diabetes, and their expression levels correlated positively with both HbA1c and BMI. These observations indicate that metabolic disturbances contribute substantially to microRNA dysregulation in patients with resistant hypertension and CHD.

Our findings extend previous observations from our cohort demonstrating associations between PBMC-derived microRNAs and T2D in patients with resistant hypertension. The consistent relationships with glycemic control observed across both studies suggest that chronic hyperglycemia influences PBMC microRNA expression beyond its effects on blood pressure alone. Given the central role of PBMCs in systemic inflammation and immune activation, the observed expression profile likely reflects the combined effects of metabolic dysregulation, chronic inflammation, and cardiovascular disease burden. These findings further support the concept that PBMC-derived microRNA expression reflects overall cardiometabolic burden rather than isolated cardiovascular pathology.

To further explore the potential clinical relevance of the identified PBMC-derived microRNAs, ROC analyses were performed to assess their ability to discriminate T2D among patients with CHD. All four microRNAs demonstrated statistically significant, albeit modest, discriminative performance, with miR-195 showing the highest AUC. The similar discriminatory performance observed across the four microRNAs is consistent with their strong co-expression pattern and suggests that they reflect a common cardiometabolic phenotype rather than independent biological processes. Nevertheless, these findings should be interpreted cautiously because of the exploratory nature of the study, the relatively small sample size, and the lack of external validation. Accordingly, further prospective studies are needed to determine whether PBMC-derived microRNAs have clinical value for cardiometabolic risk stratification in patients with RAH.

The present study has several strengths. First, microRNA expression was assessed in PBMCs, providing insight into cellular regulatory mechanisms that may more closely reflect immune and inflammatory processes involved in RAH and CHD than circulating microRNAs alone. Second, the study was conducted in a well-characterized cohort of patients with RAH who underwent comprehensive clinical and metabolic phenotyping. Finally, the simultaneous evaluation of CHD, T2D, and metabolic parameters enabled a comprehensive assessment of the interplay between cardiovascular and metabolic comorbidities and PBMC-derived microRNA expression.

## 5 Limitations

Several limitations of the present study should be acknowledged. First, the study was conducted in a relatively small cohort of patients with RAH, and some subgroup analyses, particularly those involving previous myocardial infarction, PCI, and CABG, were limited by small sample sizes. Consequently, the absence of significant differences in these analyses should be interpreted with caution.

Second, the cross-sectional design precludes conclusions regarding causality or temporal relationships between PBMC microRNA expression and the development or progression of CHD and T2D. Because microRNA expression was assessed at a single time point, dynamic changes associated with disease evolution, therapeutic interventions, or clinical outcomes could not be evaluated.

Third, microRNA expression was measured in PBMCs rather than directly in cardiovascular tissues. Although PBMC-derived microRNAs represent an accessible source of molecular biomarkers and may reflect systemic pathophysiological processes, their expression patterns may not fully correspond to those present within the myocardium, coronary vasculature, or atherosclerotic plaques.

Fourth, the analysis was limited to a predefined panel of selected microRNAs and therefore did not capture the full spectrum of microRNA dysregulation potentially associated with RAH, CHD, and metabolic abnormalities. In addition, the findings were not validated in an independent external cohort and therefore should be considered exploratory and hypothesis-generating.

Moreover, PBMCs represent a heterogeneous population of immune cells, and differences in leukocyte subset composition were not assessed. Therefore, part of the observed variability may reflect differences in PBMC cellular composition rather than changes in microRNA expression within specific cell populations.

Finally, multiple microRNAs and clinical variables were evaluated without formal adjustment for multiple comparisons, increasing the risk of type I error. Consequently, the observed associations should be considered exploratory and require confirmation in larger prospective studies with external validation.

Despite these limitations, the study provides novel evidence that CHD is associated with a distinct and coordinated PBMC microRNA signature in patients with RAH and that this expression pattern is additionally related to T2D and metabolic dysfunction.

## 6 Conclusion

The present study demonstrates that concomitant CHD is associated with a distinct PBMC-derived microRNA expression profile in patients with RAH. The coordinated upregulation of miR-195, miR-208, miR-26b, and miR-320, together with their associations with T2D, HbA1c, and BMI, indicates that PBMC-derived microRNAs reflect the complex interplay between cardiovascular and metabolic abnormalities rather than isolated hypertension. These findings support the concept that PBMC-derived microRNA profiling provides an integrated molecular signature of cardiometabolic disease burden in patients with RAH. Future prospective multicenter studies are warranted to validate these observations and to determine the prognostic significance and clinical utility of this molecular signature.

## Data Availability

The research data supporting the findings of this study are openly available in the Polish Platform of Medical Research (PPM) repository (DOI: 10.71709/wga2-rv40). The dataset “Resistant arterial hypertension, co-morbidity and selected miRNAs Database v1.0” includes anonymized clinical and molecular data relevant to miRNA expression and is accessible through the PPM research data portal.
