# Supplementary material for: MicroRNA expression profile associated with coronary heart disease in patients with resistant arterial hypertension
Source: Front Mol Biosci. 2026 Sep 10;13:1905966. doi: 10.3389/fmolb.2026.1905966 (PMC13601312; doi:10.3389/fmolb.2026.1905966)
Supplement: Supplementary file 1 [file DataSheet1.docx]

Supplementary Figure S1. Spearman correlation matrix of clinical variables and PBMC microRNA expression levels in patients with resistant arterial hypertension.

Spearman correlation coefficients (r) are displayed within each cell. Warmer colors indicate stronger positive correlations, whereas cooler colors represent negative correlations. The figure illustrates the relationships between clinical characteristics and the expression levels of the analyzed PBMC microRNAs. PBMC, peripheral blood mononuclear cell.

Supplementary Figure S2. Heatmap of correlations between PBMC microRNAs, echocardiographic parameters, and lipid profile in patients with resistant arterial hypertension.

Color intensity reflects the strength and direction of the correlation, with red indicating positive correlations and blue indicating negative correlations. PBMC, peripheral blood mononuclear cell.

Supplementary Table S1. Expression of CHD-associated PBMC microRNAs according to previous myocardial infarction, percutaneous coronary intervention, and coronary artery bypass grafting.

A. Previous myocardial infarction

| **MicroRNA** | **No MI (n = 98) Median (IQR)** | **Previous MI (n = 17) Median (IQR)** | **p value** |
| --- | --- | --- | --- |
| **miR-195** | 0.25 (−0.49–0.88) | 0.43 (−0.31–1.35) | 0.394 |
| **miR-208** | 0.83 (0.26–1.46) | 0.96 (0.43–1.95) | 0.320 |
| **miR-26b** | 0.42 (0.00–0.94) | 0.45 (0.09–1.06) | 0.695 |
| **miR-320** | −0.10 (−0.63–0.65) | 0.06 (−0.42–1.06) | 0.296 |

B. Previous PCI

| **MicroRNA** | **No PCI (n = 91) Median (IQR)** | **Previous PCI (n = 24) Median (IQR)** | **p value** |
| --- | --- | --- | --- |
| **miR-195** | 0.25 (−0.45–0.89) | 0.11 (−0.48–1.11) | 0.754 |
| **miR-208** | 0.85 (0.31–1.72) | 0.76 (0.24–1.36) | 0.850 |
| **miR-26b** | 0.46 (0.00–0.94) | 0.31 (0.05–0.94) | 0.574 |
| **miR-320** | −0.06 (−0.56–0.68) | −0.17 (−0.68–0.74) | 1.000 |

C. Previous CABG

| **MicroRNA** | **No CABG (n = 106) Median (IQR)** | **Previous CABG (n = 9) Median (IQR)** | **p value** |
| --- | --- | --- | --- |
| **miR-195** | 0.28 (−0.49–0.97) | −0.14 (−0.31–0.02) | 0.434 |
| **miR-208** | 0.85 (0.31–1.72) | 0.45 (0.21–1.02) | 0.357 |
| **miR-26b** | 0.45 (0.00–0.95) | 0.11 (0.04–0.36) | 0.290 |
| **miR-320** | −0.04 (−0.63–0.70) | −0.31 (−0.43–−0.19) | 0.514 |

Data are presented as median (interquartile range). Comparisons were performed using the Mann–Whitney U test. MI, myocardial infarction; PCI, percutaneous coronary intervention; CABG, coronary artery bypass grafting; PBMC, peripheral blood mononuclear cell.
